# Trichothecenes: From Simple to Complex Mycotoxins

**DOI:** 10.3390/toxins3070802

**Published:** 2011-07-01

**Authors:** Susan P. McCormick, April M. Stanley, Nicholas A. Stover, Nancy J. Alexander

**Affiliations:** 1 Bacterial Foodborne Pathogens and Mycology, National Center for Agricultural Utilization Research, U.S. Department of Agriculture-Agriculture Research Service, Peoria, IL 61604, USA; Email: Nancy.Alexander@ars.usda.gov; 2 Biology Department, Bradley University, Peoria, IL 61625, USA; Email: misty717@sbcglobal.net (A.M.S.); nstover@bradley.edu (N.A.S.)

**Keywords:** trichothecenes, mycotoxins, Type A, Type B, macrocyclic, d-type, t-type, toxin biosynthesis

## Abstract

As the world’s population grows, access to a safe food supply will continue to be a global priority. In recent years, the world has experienced an increase in mycotoxin contamination of grains due to climatic and agronomic changes that encourage fungal growth during cultivation. A number of the molds that are plant pathogens produce trichothecene mycotoxins, which are known to cause serious human and animal toxicoses. This review covers the types of trichothecenes, their complexity, and proposed biosynthetic pathways of trichothecenes.

## 1. Introduction

Fungi produce a large number of metabolites that are not essential for life, but may provide the fungus with an ecological advantage in certain environments. Such metabolites are referred to as secondary metabolites. Fungal secondary metabolites include plant growth regulators (e.g., gibberellins), pharmaceutically useful compounds (e.g., penicillin, lovastatin), pigments (e.g., carotenoids), and mycotoxins (e.g., trichothecenes, fumonisins, aflatoxins, ochratoxins) [1]. Mycotoxins may accumulate in infected crop plants, and upon ingestion, lead to the development of diseases (mycotoxicoses) in humans and animals [2]. Trichothecenes are one of the major classes of mycotoxins, causing a significant economic impact on cereal and grain crops each year [3,4]. An understanding of the pathogenicity of trichothecene-producing fungi and the regulation of trichothecene biosynthesis has become increasingly important to combat their spread throughout North America and other areas worldwide.

Trichothecene-producing genera include *Fusarium*, *Myrothecium*, *Spicellum*, *Stachybotrys*, *Cephalosporium*, *Trichoderma*, and *Trichothecium* [5]. These fungi, of the order Hypocreales, are found throughout the world and are adapted for colonization and growth on substrates with a wide range of moisture availability and nutrient content. Within the genus *Fusarium*, some species are important plant pathogens and are the causes of wilts, blights, and ear rots in grains, especially wheat, barley, oats and maize [4,6]. *Myrothecium* species are pathogens of muskmelon and tomato [7,8]. *Stachybotrys* is found in a variety of commodities and more recently, has been found to be a significant indoor environmental contaminant that has been correlated with damp building-related illnesses [9]. *Trichoderma* species are commonly found in the soil and have been associated with diseases of mushrooms and grapes [10]. *Trichothecium* species are commonly found in the soil and on decaying organic material [11]. 

Early toxicity studies showed that trichothecenes inhibit eukaryotic protein synthesis, specifically by preventing peptide bond formation at the peptidyl transferase center of the 60S ribosomal subunit. This inhibition typically affects polypeptide chain initiation or elongation, although polypeptide chain termination may also be inhibited [12,13,14,15]. Trichothecenes were later shown to inhibit mitochondrial protein synthesis [16,17] and to interact with protein sulfhydryl groups [18]. The activity of trichothecenes eventually produces harmful levels of oxidative stress due to generation of free radicals [19,20].

Trichothecenes are small, amphipathic molecules that can move passively across cell membranes [21,22]. They are easily absorbed via the integumentary and gastrointestinal systems, allowing for a rapid effect of ingested trichothecenes on rapidly proliferating tissues [22]. Exposure to these toxins can cause feed refusal, immunological problems, vomiting, skin dermatitis, and hemorrhagic lesions [15,23]. They are also phytotoxic and can cause chlorosis, inhibition of root elongation, and dwarfism [14,24], and act as a virulence factor in wheat head scab [25,26,27].

**Figure 1:**
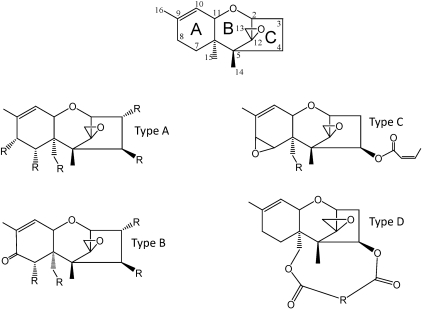
Classification of trichothecene structures. EPT (12,13-epoxytrichothec-9-ene); R groups may be H, OH, OAcyl, or variations in the macrolide chain.

Trichothecenes are a family of over 200 toxins with a common tricyclic 12,13-epoxytrichothec-9-ene (EPT) core structure (Figure 1) [5,28]. They have been classified into four groups (Types A, B, C, and D) based on the substitution pattern of EPT (Figure 1) [29,30]. Types A, B and C can be differentiated based on the substitution at the C-8 position. Type A trichothecenes include compounds that have a hydroxyl group at C-8 (e.g., neosolaniol), an ester function at C-8 (e.g., T-2 toxin), or no oxygen substitution at C-8 (e.g., trichodermin, 4,15-diacetoxyscirpenol, and harzianum A). Type B trichothecenes have a keto (carbonyl) function at C-8 (e.g., nivalenol, deoxynivalenol, and trichothecin). In *Fusarium*, Type B trichothecenes typically have a C-7 hydroxyl group, but this structural feature is not present in other genera.

Type C trichothecenes have a C-7/C-8 epoxide (e.g., crotocin). Type D trichothecenes have an additional ring linking the C-4 and C-15 position (e.g., roridin A, verrucarin A, satratoxin H). Although this classification system has continued to be used [31,32,33], there are other structural features that are not accounted for with this system. For example, all *Fusarium* trichothecenes (including Type A and Type B) have an oxygen function (*i.e.*, a hydroxyl or an acetyl group) at C-3. Trichothecenes produced by *Trichoderma, Trichothecium, Myrothecium* or *Stachybotrys* (including Types A, B, C and D) lack an oxygen function at the C-3 position. With the current ability to study the genetic control of trichothecene biosynthesis, a genetic approach to trichothecene classification is possible. 

## 2. Trichothecene Biosynthesis in *Fusarium*

**Figure 2:**
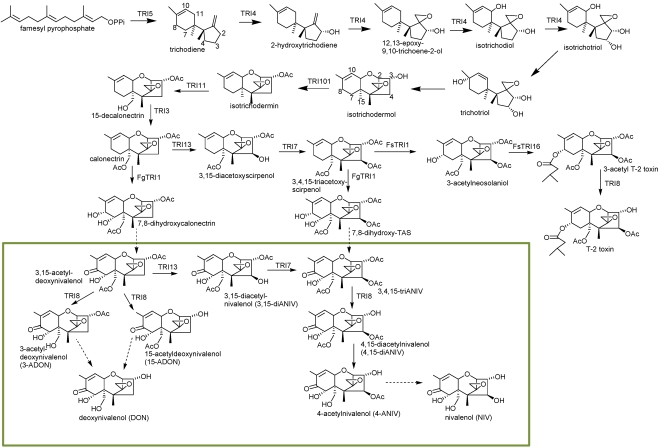
Proposed trichothecene biosynthetic pathway in *Fusarium*. Genes encoding an enzymatic step are identified near the arrow indicating the step. Dashed arrows indicate steps for which a gene has not been assigned. Green box indentifies Type B trichothecenes.

Biosynthesis of *Fusarium* trichothecenes begins with the cyclization of farnesyl pyrophosphate, a primary metabolic intermediate, to form trichodiene. The terpene cyclase trichodiene synthase (Tri5) that catalyzes this reaction and the gene that encodes it (*TRI5*) were first characterized in a T-2 toxin (Type A trichothecene) producing strain of *Fusarium sporotrichioides* [34,35] (for the *Fusarium* trichothecene biosynthetic pathways, see Figure 2). Trichodiene undergoes a series of oxygenations catalyzed by a cytochrome P450 monooxygenase encoded by *TRI4* [36]. In *Fusarium* species, *TRI4* controls the addition of four oxygens at C-2, C-3, C-11, and the C-12, C-13-epoxide to form the intermediate isotrichotriol [37].

**Figure 3:**
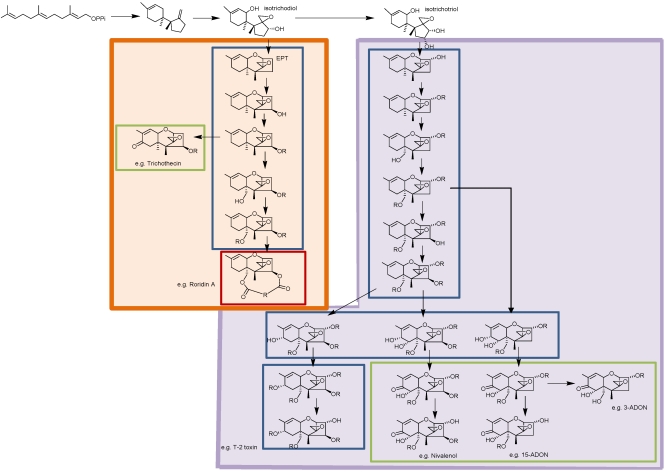
Proposed trichothecene biosynthetic pathways illustrating the divergence into the d-type (from isotrichodiol) (orange box) and the t-type (from isotrichotriol) (violet box) trichothecenes. Blue boxes indicate Type A trichothecenes; green boxes indicate Type B trichothecenes; red box indicates Type D trichothecene.

Isotrichotriol undergoes a non-enzymatic isomerization and cyclization to form isotrichodermol (=3α-hydroxy EPT) [38]. During this process, the oxygen at the C-2 position becomes the pyran ring oxygen and the hydroxyl group at C-11 is lost. More complex Type A trichothecenes are built by modifying isotrichodermol through a series of paired hydroxylation (–OH) and acetylation or acylation (–OR) steps (Figure 3).

Isotrichodermol (C-3 –OH) is converted to isotrichodermin (C-3 –OR) (Figure 2) by an acetyltransferase encoded by *TRI101* [39]. This step effectively reduces the toxicity of *Fusarium* trichothecenes, thereby serving as a mechanism for the self-protection of the trichothecene-producing organism [40]. A second hydroxyl group is added to C-15 (controlled by *TRI11*) [41], which is subsequently acetylated under the control of *TRI3* [42,43]. A third hydroxyl group is added at C-4 (controlled by *TRI13*) [44,45], which is subsequently acetylated under the control of *TRI7* [44]. In *F. sporotrichioides*, a Type A producer, a fourth hydroxyl group is added to C-8 (controlled by *TRI1*), followed by the addition of an isovaleryl moiety controlled by *TRI16*. Finally, the acetyl group is removed from the C-3 position by an esterase (controlled by *TRI8*) to produce T-2 toxin [46].

Biosynthesis of *Fusarium* Type B trichothecenes, e.g., 15-ADON or 4,15-diANIV, follows a pathway similar to that of Type A trichothecenes, with paired hydroxylations and acetylations at C-3 and C-15, or at C-3, C-15 and C-4 (Figure 2 and Figure 3). However, rather than *TRI1* controlling the final hydroxylation at C-8 as in Type A-producing strains, *TRI1* in Type B-producing strains controls the addition of hydroxyl groups at both the C-7 and C-8 positions [47,48]. The C-8 hydroxyl group is then converted to a keto function for which the genetic control has not been fully characterized. In *Fusarium*, presence of a C-7 hydroxyl group is correlated with the conversion of the C-8 hydroxyl group to a keto function. The final step in *Fusarium* Type B trichothecene biosynthesis is the removal of the C-3 acetyl group, or the C-15 acetyl group, by an esterase encoded by *TRI8*. Differential activity of this esterase, as defined by the DNA sequence, determines production of either 3-ADON or 15-ADON chemotypes in *F. graminearum* [49]. 

## 3. Trichothecene Biosynthesis in Other Genera

Biosynthesis of trichothecenes in other genera also begins with the cyclization of farnesyl pyrophosphate to form trichodiene (Figure 3). Homologs of *Fusarium TRI5* that control this step have been identified in *Myrothecium roridum* [50], *Trichothecium roseum* [51], *Trichoderma harzianum* [52], and *T. brevicompactum* [53]. 

Most trichothecenes produced by *Myrothecium*, *Trichothecium*, *Trichoderma* and other genera lack a C-3 hydroxyl group. Homologs of *Fusarium TRI4* have been identified in *Myrothecium roridum* [50] and *Trichothecium roseum* [51] and in these genera, *TRI4 * controls the addition of only three, rather than four, oxygens: hydroxyl groups at C-2 and C-11, and the C-12, C-13 epoxide, converting trichodiene into isotrichodiol [51,54]. However, *T. roseum* produces two types of trichothecenes, those that lack a C-3 oxygen function (e.g., trichothecin), and those that have a C-3 hydroxyl group (e.g., trichothecinol A) [5,55]. It is not known if the C-3 hydroxyl group is added prior to cyclization or exactly which gene controls this oxygenation step. 

Non-enzymatic isomerization and cyclization of isotrichodiol, with loss of a hydroxyl group, forms 12,13-epoxytrichothec-9-ene (EPT). More complex trichothecenes are formed from EPT by a series of hydroxylation (–OH) and esterification (–OR) reactions (Figure 3). Although the genes and enzymes for these steps have not been characterized, the types of genes required can be discerned from the structures of the toxins. The relatively simple Type A trichothecene, trichodermin, produced by *Trichoderma brevicompactum*, requires only the addition of a single hydroxyl group at C-4 by a P450 oxygenase (–OH), followed by an acetylation at that position (–OR). Biosynthesis of harzianum A, a Type A trichothecene produced by *T. arundinacium*, requires C-4 hydroxylation and esterification with a polyketide side chain. This type of substitution necessitates the involvement of a polyketide synthase as well as a transferase. Biosynthesis of trichothecin, a Type B trichothecene produced by *Trichothecium roseum*, is likely formed from EPT also. In this case, EPT undergoes hydroxylation at C-4 (–OH) followed by esterification with a butyryl group (–OR), which is then followed by a second hydroxylation at C-8 (–OH). Conversion of the C-8 hydroxyl group to a keto produces trichothecin, a type B trichothecene group. Although *Fusarium* Type B trichothecenes typically have an 8-oxo, C-7 hydroxyl substitution pattern, Type B trichothecenes in other genera lack a C-7 hydroxyl group. 

Macrocyclic trichothecenes lack a C-3 oxygen function, indicating that their biosynthesis proceeds from isotrichodiol and EPT. To build these compounds, the initial hydroxylation and esterification of a polyketide at C-4 is followed by the addition of a hydroxyl group (e.g., trichoverrol A) and then a polyketide ester at C-15 (e.g., trichoverrin A). Condensation of the C-4 and C-15 polyketide chains would result in a Type D macrocyclic trichothecene [10].

## 4. Genetic Approach to Trichothecene Classification

In fungi, genes responsible for the biosynthesis of secondary metabolites are typically located next to one another in gene clusters [1,50,56,57]. In *Fusarium*, trichothecene genes (*TRI*) are found at three loci [58,59]: a 26-kb core *TRI* cluster containing most of the genes that control trichothecene biosynthesis [60]; a two-gene *TRI1-TRI16* cluster [61,62]; and the *TRI101* locus [63]. In addition, *TRI15*, which appears to negatively control trichothecene biosynthesis, is located outside the core cluster [64] and may be part of a siderophore biosynthesis gene cluster [65]. In some species (e.g., *F. equiseti*), *TRI1* and *TRI101* are inserted into the core cluster suggesting a complex evolutionary history of the trichothecene genes [59]. The functions of most of these genes in the biosynthesis, regulation of biosynthesis and transport of trichothecenes, have been characterized in *Fusarium* and reviewed [58,66]. The identification of genes and their proteins now enable us to view how genetic changes lead to different types of trichothecenes. 

Table 1 shows the chemical classification of nineteen representative trichothecenes by four different methods. Although the Type A, B, C, and D classification is commonly used, other classifications have been suggested. Trichothecenes can be classified as simple or macrocyclic. Biosynthesis of both simple trichothecenes (Type A, B, or C) and macrocyclic trichothecenes (Type D) is actually via Type A trichothecene intermediates (Figure 3). Grove [67] focused on conformational differences and reactivity of functional groups, and designated three groups of simple trichothecenes. Group I contains trichothecenes that have substitutions only on ring C (see Figure 1 for ring identification), e.g., trichodermin, isotrichodermin; Group II contains trichothecenes which have additional substituents in ring A, e.g., T-2 toxin and 7,8-dihydroxycalonectrin (Figure 1 and Figure 2); and Group III contains trichothecenes that have a keto at C-8, e.g., DON or trichothecin, the same criteria for Type B trichothecenes.

Kimura *et al.* [68] proposed dividing trichothecenes into two groups based on whether they are derived from isotrichodiol (d-type) or isotrichotriol (t-type) (Figure 3). This classification turns out to be genetically based on functional differences in *TRI4*, which controls the addition of either 4 oxygens, as in *Fusarium* sp. (t-type), or 3 oxygens as in other genera (d-type) [37,51,54] (Figure 3). Phylogenetic analyses may help to determine how d-type and t-type trichothecenes evolved.

**Table 1:** Classification of selected trichothecenes.

Trichothecene	Simple or Macrocyclic (S or M)	Type ^1^	Group ^2^	Type ^3^
1 trichodermol	S	A	I	d
2 trichodermin	S	A	I	d
3 4,15-Diacetoxyscirpenol (DAS)	S	A		t
4 neosolaniol	S	A	II	t
5 T-2 toxin	S	A	II	t
6 isotrichodermol	S	A	I	t
7 calonectrin	S	A		t
8 7,8-dihydroxy calonectrin	S	A	II	t
9 harzianum A	S	A		d
10 nivalenol (NIV)	S	B	III	t
11 deoxynivalenol (DON)	S	B	III	t
12 fusarenon-X	S	B	III	t
13 trichothecin	S	B	III	d
14 trichothecinol A	S	B	III	t
15 crotocin	S	C		d
16 satratoxin H	M	D		d
17 roridin A	M	D		d
18 baccharin	M	D		d
19 verrucarin A	M	D		d

^1^ Based on presence of C-8 keto group (Type B), C-7, C-8 epoxy (Type C), ring connecting C-4 and C-15 (Type D) [29]; ^2^ Based on substitutions in the A and C ring [67]; ^3^ Based on presence (t-type)or absence (d-type) of C-3 oxygen function [40].

Relating toxicity of individual trichothecenes based on any of these classification systems is not always straightforward. Macrocyclic trichothecenes (e.g., roridins, verrucarins and satratoxins) are considered to be some of the most toxic trichothecenes and are generally more toxic than simple trichothecenes [9,69]. Type A trichothecenes such as DAS and T-2 toxin are generally more cytotoxic than Type B trichothecenes such as DON [29]. Animal toxicity increases with increasing oxygenation of EPT [70]. Testing of *Fusarium* trichothecenes on the model plant system *Chlamydomonas* showed that both Type A and Type B C-3 acetylated trichothecenes are much less toxic than the corresponding C-3 hydroxyl trichothecenes [71]. In *Fusarium*, one gene, *TRI101*, controls this change in toxicity. A more extensive survey of trichothecenes for phytotoxicity showed that there can be wide variation in the phytotoxicity within Type A trichothecenes and that some are relatively non-toxic [72]. In addition, this study found that small structural changes can result in large changes in toxicity and that some Type B trichothecenes are relatively more toxic than Type A trichothecenes. Therefore, the degree of toxicity of trichothecenes does not necessarily correlate with chemical classification. 

## 5. Conclusions

In recent years, the world has experienced an increase in awareness of mycotoxin contamination of grains, building materials, and air-handling systems. Many of these problems are caused by trichothecenes, which range from those with relatively simple structures to those with more complex, macrocyclic structures. Gene identification has begun to clarify how trichothecenes are made. As more genetic sequence data becomes available, phylogenetic analyses may lead to a better understanding of the evolution of trichothecene-producing fungi. Phylogenetic work on the trichothecene gene cluster indicates that, through time, there has been consolidation of *TRI* genes into fewer loci in some fusaria while three distinct *TRI* loci have been maintained in other fusaria [59]. Continued studies will help to clarify how evolution affects mycotoxin-producing genes. Using gene sequences from one organism also helps in the identification of trichothecene genes and pathways in other organisms, such as using *Fusarium* genes to identify those in *Trichoderma* (Santiago Gutiérrez, personal communication). Studying fungal populations and genetic linkages will also bring a better understanding of how pathogenic fungi spread throughout the world [73,74,75,76]. Comparison of gene sequences from expanding databases may also contribute to identification of trichothecene-related genes that may lead to new control strategies to reduce both crop diseases caused by the fungi and the mycotoxin contamination problems associated with these diseases. The combination of chemical and genetic analysis of trichothecene biosynthesis may lead to a better understanding of the evolution and phylogenetics of the trichothecenes. 
